# Pneumatic piston hydrostatic bioreactor for cartilage tissue engineering

**DOI:** 10.1080/10739149.2022.2124418

**Published:** 2022-09-20

**Authors:** J. Hallas, A. J. Janvier, K. F. Hoettges, J. R. Henstock

**Affiliations:** a Department of Musculoskeletal and Ageing Science, Institute of Life Course and Medical Sciences, University of Liverpool, Liverpool, L7 8TX, UK; b The Medical Research Council Versus Arthritis Centre for Integrated Research into Musculoskeletal Ageing (CIMA), University of Liverpool, UK; c Department of Electrical Engineering and Electronics, University of Liverpool, Liverpool, UK

**Keywords:** Bioreactor, cartilage, chondrocytes, hydrostatic pressure, mechanobiology

## Abstract

During exercise, mechanical loads from the body are transduced into interstitial fluid pressure changes which are sensed as dynamic hydrostatic forces by cells in cartilage. The effects of these loading forces in health and disease are of interest to biologists, but the availability of affordable equipment for *in vitro* experimentation is an obstacle to research progress. Here, we report the development of a cost-effective hydropneumatic bioreactor system for research in mechanobiology. The bioreactor was assembled from readily available components (a closed-loop stepped motor and pneumatic actuator) and a minimal number of easily-machined crankshaft parts, whilst the cell culture chambers were custom designed by the biologists using CAD and entirely 3 D printed in PLA. The bioreactor system was shown to be capable of providing cyclic pulsed pressure waves at a user-defined amplitude and frequency ranging from 0 to 400 kPa and up to 3.5 Hz, which are physiologically relevant for cartilage. Tissue engineered cartilage was created from primary human chondrocytes and cultured in the bioreactor for five days with three hours/day cyclic pressure (300 kPa at 1 Hz), simulating moderate physical exercise. Bioreactor-stimulated chondrocytes significantly increased their metabolic activity (by 21%) and glycosaminoglycan synthesis (by 24%), demonstrating effective cellular transduction of mechanosensing. Our Open Design approach focused on using ‘off-the-shelf’ pneumatic hardware and connectors, open source software and in-house 3 D printing of bespoke cell culture containers to resolve long-standing problems in the availability of affordable bioreactors for laboratory research.

## Introduction

Mechanical forces deriving from exercise are an essential stimulus for maintaining biological homeostasis in functional joint cartilage, whilst aberrant mechanical loading plays a role in degenerative conditions such as osteoarthritis.^[^^1^^,^^2^^]^ The musculoskeletal system is composed of cells and tissues that are force responsive, and physical loading of bone, cartilage, muscle, and tendon results in adaptation of the tissue for increased resilience.^[^^3^^]^ Stronger osteochondral tissues are formed in response to environmental demands due to an increase in cell activity that produces extracellular matrix molecules such as collagenous proteins and glycosaminoglycans.^[^^4^^,^^5^^]^ In combination with other cues, including systemic (endocrine) and local biochemical signaling (e.g., growth factors and cytokines), mechanical forces play an important role in enabling the cell to sense its environment. Mechanical forces acting on a cell are converted into changes in intracellular signaling pathways by mechanotransduction events, including mechanosensitive ion channel activation, integrin-mediated signaling between the extracellular matrix and the cytoskeleton, and an array of other mechanically-linked processes. This connection between mechanical stimuli and changes in cell response is of interest to a range of disciplines including in biomedical tissue engineering strategies to create replacement graft tissues formed from hydrogel-encapsulated cells cultured in dynamic growth environments. ^[^^6–9^^]^

Of all the tissues in the musculoskeletal system, the mechanobiology of articular cartilage is of particular interest due to the increasing prevalence of osteoarthritis in the global population.^[^^10^^]^ More than one in seven people worldwide (654 million) suffer from osteoarthritis, a serious, debilitating and untreatable disease which severely impacts quality of life and ability to work.^[^^11^^]^ No pharmaceuticals are available to prevent the progression of osteoarthritis, and whilst total joint replacement is an effective end stage treatment, it follows years or even decades of pain and restricted mobility. Understanding the intracellular mechanisms by which cartilage is maintained in homeostasis by exercise is fundamental to understanding and treating osteoarthritis, and to do so we must develop appropriate tools for mechanobiology research under controlled *in vitro* experimental conditions.^[^^2^^,^^12^^,^^13^^]^ Similarly, understanding the role of mechanical force in healthy cartilage growth may enable improved strategies for arthroscopic repair and tissue engineering.^[^^6^^]^

Bioreactors have been developed to investigate the cell response to a variety of different mechanical forces, depending on the predominant type of mechanical stress in each tissue.^[^^8^^,^^14^^]^ In cartilage during routine locomotion, bodyweight generates periodic confined compressions of the fluid-filled synovium and the highly hydrated articular cartilage, resulting in the generation of cyclic hydrostatic forces.^[^^15–17^^]^ It has therefore been theorized that the principal cell type in cartilage, the chondrocyte, has evolved to be particularly sensitive to this type of mechanical loading force.^[^^15^^]^ Bioreactor companies have developed a variety of approaches to generate physiological levels of hydrostatic pressure *in vitro*, which aim to mimic the type and range of loading experienced by chondrocytes under controlled experimental conditions, but these devices are often expensive and out of reach for many research groups.^[^^18–20^^]^ In particular, the commercial availability of pulsatile hydrostatic pressure bioreactors remains extremely limited, which has had a negative impact on the volume of reproducible research.^[^^20^^]^ There is a requirement for more affordable, versatile, and adaptable hardware which can be easily modified by the researcher to suit a wider range of research applications using fabrication techniques which are now widely available, such as 3 D printing.^[^^14^^]^

In this report, we describe the design, construction and testing of a pneumatic-hydrostatic bioreactor device that applies pulsed pressure to stimulate the growth of human chondrocytes in a bioengineered cartilage tissue. The bioreactor design features a high-torque stepper motor driving a reciprocating pneumatic piston via a convenient user-controlled Windows interface. The design criteria were to apply pulses of pressure at a pre-determined frequency, magnitude and duration to a gas-tight container containing samples of tissue engineered cartilage. Our objective was to develop a low-cost and easily reproducible bioreactor by using commercially available hardware, open source software and 3 D printing.

## Materials and methods

### Bioreactor materials

**Figure 1.**
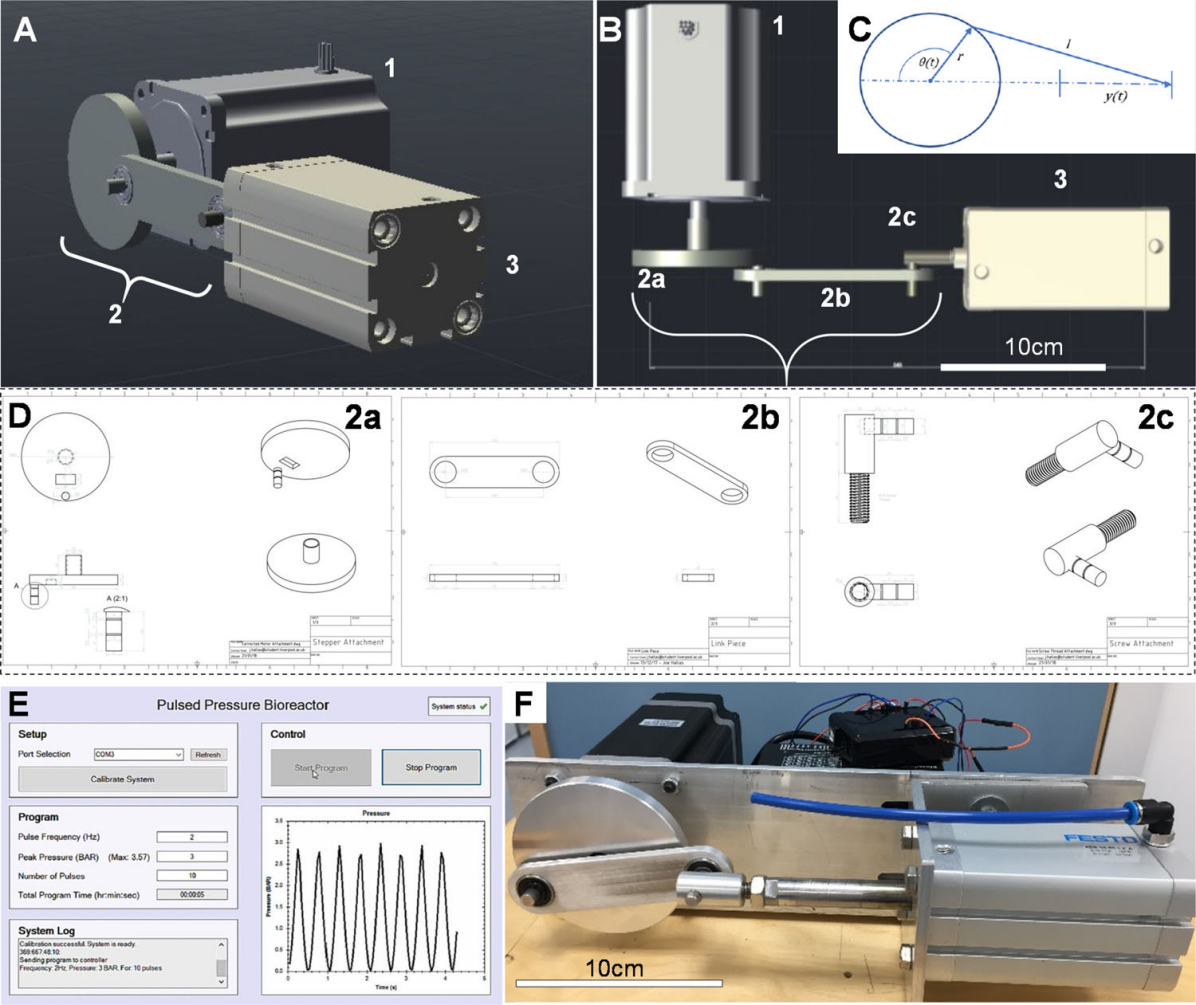
**(A, B)** Pneumatic pulsed pressure bioreactor composed of a (**1**) 13 N·m closed-loop bipolar stepper motor with a (**2**) 40 mm radius crankshaft mechanism driving a (**3**) 50 mm bore 157 ml pneumatic actuator piston with an 80 mm stroke. (**C**) The linear piston displacement was calculated using (*t*)≈*r*(1 − cos(θ(*t*))) with a resolution of 0.36°/step at the motor. (**D**) Three components were designed and machined in stainless steel to enable a robust mechanical connection between the stepper motor and the pneumatic piston, comprising a (**2a**) crankshaft, (**2 b**) a linkage rod, and (**2c**) a screw attachment which directly affixed to the piston actuator rod. (**E**) The bioreactor was driven by a closed-loop stepper driver and controlled by an Arduino UNO microcontroller, whilst a real-time user interface was created in C# for Windows for control over experimental variables (pressure frequency and maximum load, duration/number of cycles) *via* USB to the Arduino. A 400 kPa analogue sensor was used to provide real-time feedback of the pressure created in the chamber, and a Hall effect sensor used to control positional accuracy of the shaft. (**F**) When tested, the bioreactor was capable of outputting pulses of air from the cylinder at frequencies up to 3.5 Hz and pressures of up to 400 kPa. Scale bar is 10 cm.

The design parameters of the bioreactor hardware are shown in Table 1, and a schematic of the bioreactor is shown in Figure 1. A NEMA 34 closed loop bipolar stepper motor (13 N m holding torque, 14 mm diameter shaft) with in-built 1000 cpr (counts per revolution) encoder, and a CL86T motor driver (0 − 8.2 A with microstepping) were purchased from StepperOnline (UK). The motor was powered via a 150 W, 36 V, 4.16 A desktop power supply (RS components). A Festo double acting pneumatic actuator (10 bar maximum, 157 ml volume, 50 mm bore, 80 mm stroke, ADN Series 50-80-A-P-A) was purchased from RS Components. All bespoke parts were designed using AutoCAD and either machined in house from 6 mm aluminum sheet or 3 D printed in PLA using an Ultimaker 2 3 D printer. 6 mm diameter screw in, push fit pneumatic elbow connectors and 6 mm polypropylene air hose were used to connect the bioreactor hardware to the cell culture chamber. The costs for each bioreactor component (UK prices, 2022) are shown in Supplementary Table S1. The engineering calculations used for parameterization are available in Appendix A in the supplementary material.

**Table 1.** Overall design parameters for the bioreactor.

Parameter	Value
Cell culture chamber volume	52.3 ml
Pressure achieved	300 kPa
Maximum force acting on piston	392.90 N
Maximum torque acting on motor shaft	7.34 Nm
Location of maximum torque	134.2°

### Motor driver

The 3 digital input and 2 digital output pins of the motor driver were controlled and read by an Arduino microcontroller (Supplementary Table S2). Negative pins were connected to a common ground provided by the Arduino. The encoder signal was transmitted via a 15-pin DB male plug from the motor. The motor was driven using 1000 steps per revolution which corresponds to a movement of 0.36° per step (Figure 1C).

### Transmission

**Figure 2.**
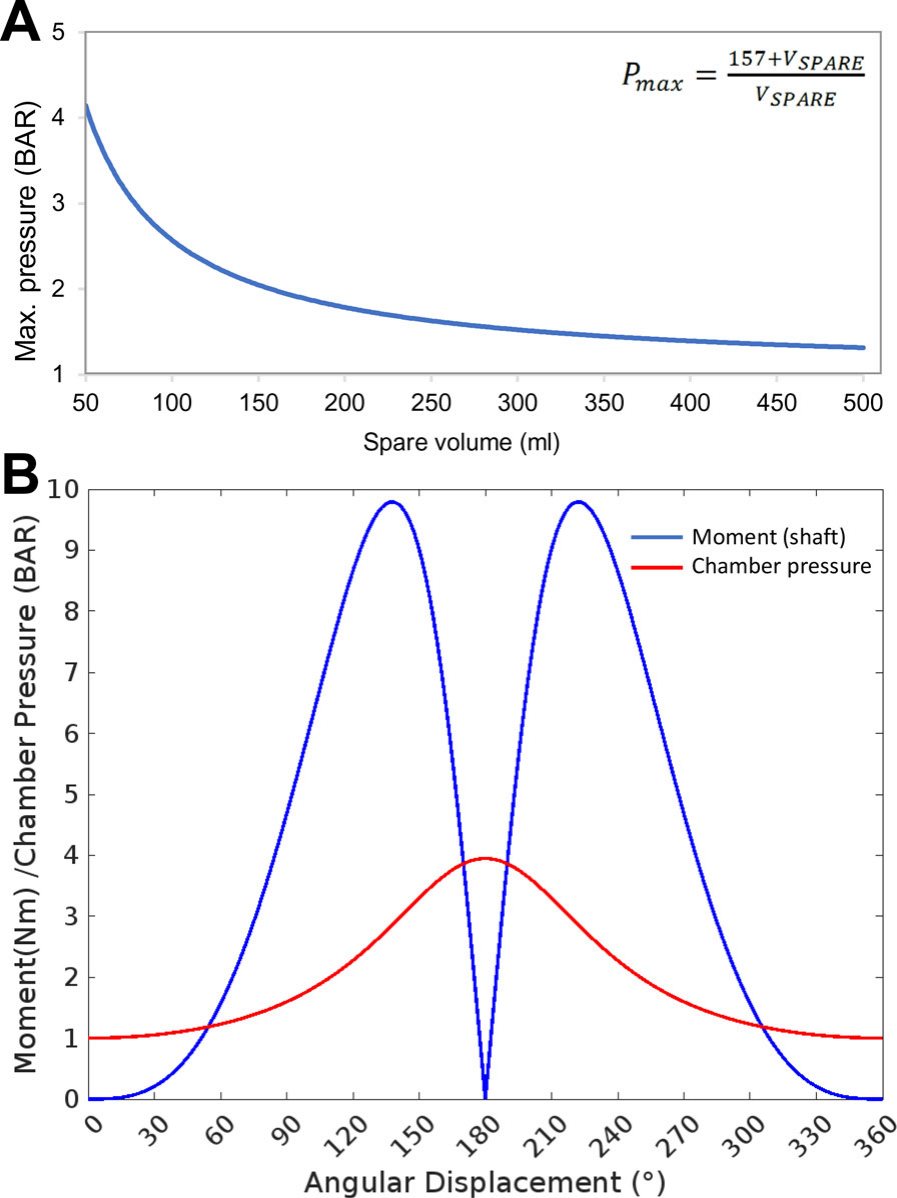
Parameterization of the bioreactor. Determining the operational parameters involved establishing the relationship between the compressible air volume in the system, which was a variable based on the liquid/cell volume occupying the cell culture chamber, and the mechanical moment applied by the motor-piston assembly. (**A**) The relationship between maximum chamber pressure and spare volume in the system was calculated and plotted. (**B**) The maximum achievable chamber pressure and resulting torque/moment acting on the motor shaft through a full rotation was calculated based on attachment of the apparatus to an empty 57.3 ml bioreactor chamber.

The transmission body of the system was created from 6 mm aluminum sheet and machined as three separate modular components: (a) rotational connection to the motor shaft (crank), (b) linkage connection with a pivot point at each end, and (c) a rod-end connection to the pneumatic cylinder piston (Figure 1D). A secure connection to the motor was designed with a 5 mm thick slot to surround the 14 mm diameter motor shaft with a grub screw. The part was designed so that the crank radius was 40 mm. The connecting shaft was 8 mm diameter and designed to include spaces for two 0.8 mm circlips to hold the joining piece in place laterally. The pneumatic cylinder piston was connected to the system body by a 10 mm diameter female screw thread. An M10 screw thread was connected to a short cylindrical section of a 15 mm diameter cylinder which held a connecting shaft of 8 mm diameter to connect to the joining piece. This connecting shaft accommodated two 0.8 mm circlips. To link the connection pieces together, two 22 mm diameter rotational ball bearings were used to minimize the friction in the system and reduce the load acting against the motor. A 6 mm thick aluminum sheet was used as a chassis frame to align the motor and pneumatic cylinder. The sheet was fixed across the front plate of the motor and using an aluminum clamp on the pneumatic cylinder to allow for small horizontal adjustments to be made. Calculations based on the forces, pressures, and volumes of the apparatus were used to parameterize force displacement curves (Figure 2).

### Microcontroller

An Arduino Uno (Atmega 238p) was used to control the motor, take inputs from other sensors and components in the system, and to synchronize this process with instructions sent by the user from the PC interface (code available in Appendix C in the supplementary material). Simple instructions were established to enable the motor to be moved 3 steps (1 degree) either clockwise or anti-clockwise for manual calibration. To calibrate using the distance sensor, the Arduino continued to read the distance from the sensor until a value of 8 cm or below was detected, the motor was then rotated 420 steps to return the system to its correct starting state. The complete wiring diagram is shown in supplementary Figure S1.

### Ultrasonic distance sensor

An HC-SR04 40 kHz ultrasonic sensor (RS Components) was used to detect piston displacement to an accuracy of within 3 mm for initial testing and on-going calibration of the piston start point. The sensor was placed below the point at which the linking piece passes over at maximum displacement (i.e., fully compressed piston) and measured a change in distance 7 cm above the sensor. At the point of maximum displacement (maximum pressure), the pressure was recorded so that the user may know the maximum available pressure. Following this, the motor rotated 180° back to the starting position, and the pressure in the chamber was read to ensure return to 1 bar, verifying that the chamber was gas-tight and the system calibrated in preparation for use.

### Software development and user interface

A Windows user interface for the system was developed using C# Windows Forms (Figure 1E). The interface was designed using panels that split the window into sub-sections for clarity and ease of use. The ‘Setup’ panel allowed the user to select the serial port that the bioreactor was connected to. The drop-down box was automatically populated with the options detected and featured a refresh button for if the user had failed to connect the system before opening the program. Once calibrated, the maximum pressure available to the system was displayed within the ‘Program’ panel. This value was sent from the Arduino as a string over serial port and processed by the interface. All other actions were disabled before the system is calibrated to ensure that the system could only run when fully and safely prepared. Once calibrated, the start and stop buttons were enabled. The program values were entered within the ‘Program’ panel. The ‘total program time’ was calculated based on the number of pulses and frequency selected, and automatically formatted into hours, minutes, and seconds. The system run start was accompanied by a graphical readout showing the expected pressure output in the bottom-right hand panel. A system log box continuously updated the user on the current processes, along with any error messages that may be generated by the interface or the Arduino itself. In the top right-hand corner, a system status symbol was shown. Potential errors include disconnection of USB cable (and hence loss of serial port), current/voltage issues (an error message sent from the Arduino), or invalid data being returned from the system.

### Cell culture chamber

**Figure 3.**
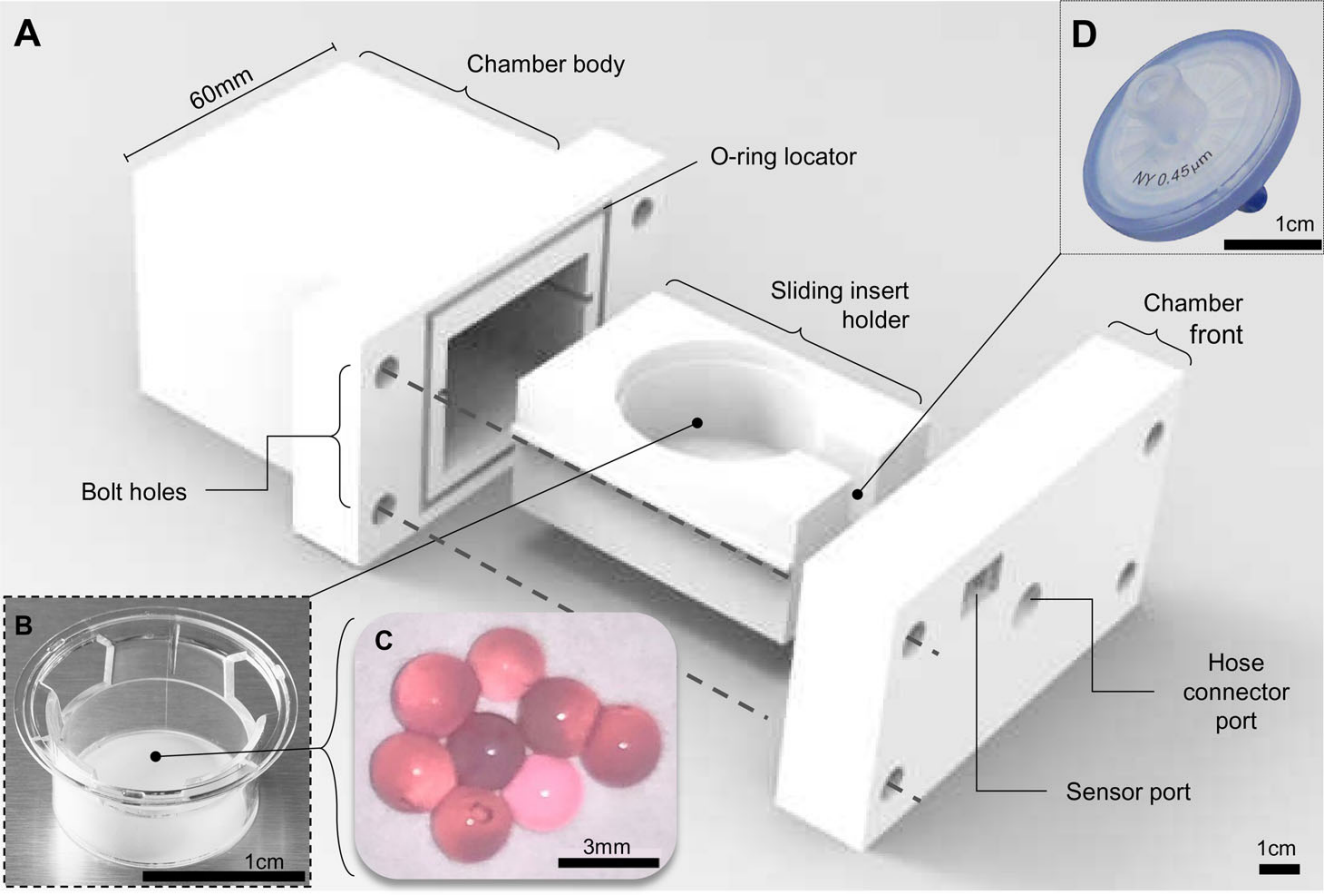
The 3 D printed cell culture bioreactor pressure chamber. (**A**) A 3 D printed bioreactor culture chamber was designed with a removable sliding tray to hold (**B**) a standard transwell insert containing (**C**) microspheres of tissue engineered cartilage which could be assembled in a sterile flow hood and made gas-tight using four simple bolts and a 4 mm neoprene O-ring as a gasket. The chamber was (**D**) connected to the main bioreactor pressure outlet via a pressure hose with an inline sterile 25 mm (0.4 μm) syringe filter to sterilize the input air which fitted into a recess on the inside of the chamber front. The scale bars in Figure 3A, B, and D are 1 cm and 3 mm in Figure 3C.

A cell culture chamber was designed and 3 D printed in polylactic acid (PLA) to contain the cells in a pressurized environment, comprising a main chamber body (external housing, dimensions L: 70 mm x W: 60 mm x H: 50 mm), slide-out compartment to contain the live culture, and a front panel containing the push fit port connection to the pneumatic piston (M6 Festo push fit pneumatic connector) with an inline air filter (0.4 μm 13 mm syringe capsule filter, Corning) and a pressure sensor (Figure 3). A standard commercially available transwell insert (Corning) holding the actual cell cultures was placed in a sliding internal compartment. The chamber was assembled in a sterile Class II biosafety cabinet, and sealed via a 4 mm neoprene O-ring between the chamber and the front panel with four M4 bolts with washers to create a pressurisable chamber. The chamber was removed from the biosafety cabinet and attached to the piston via a 6 mm push-in Festo pneumatic adapter and polypropylene air hose. Subtracting the volume occupied by the transwell and cell culture media, the chamber had a gas volume of 57.3 ml, and so the required cylinder compression was calculated based on these values (Figure 2).

### Chondrocyte cell culture and tissue engineered cartilage

Human chondrocytes were isolated from knee cartilage donated from total knee replacement via the Liverpool Musculoskeletal Biobank. Undamaged cartilage was dissected away from the joint and placed in 1 mg/ml collagenase solution in DMEM media at 37 °C for 24 hours. Chondrocytes were recovered by passing the digested cartilage through a 100 µm cell sieve and pelleted by centrifugation at 400 RCF for 5 minutes. The chondrocytes were cultured in T-flasks until 70% confluent and trypsinised, recovered again by centrifugation, and encapsulated in 2% w/v alginate at 3 x 10^5^ cells per ml using a droplet encapsulation method in a 200 mM calcium chloride bath to generate 64 replicate spherical microtissues measuring approximately 6 mm in diameter (Figure 3C).^[^^21^^]^ Encapsulated chondrocytes were cultured in chondrogenic media (low glucose DMEM, 1x insulin-transferrin-selenium solution, 10^−8^ dexamethasone, L-proline, 1x penicillin-streptomycin-amphotericin) for 5 days either under consistently static incubator conditions, or with periodic stimulation in the bioreactor chamber. The bioreactor stimulated group received 300 kPa peak pressure at 1 Hz for 3 h/day over 5 days, and were returned to regular incubator culture for the remaining 19 h/day. After 5 days, the chondrocytes were recovered from the alginate matrix using a dissociation buffer (155 mM sodium citrate, 55 mM sodium chloride) to de-crosslink the alginate, with each individual hydrogel sphere immersed in 1 ml buffer for 15 minutes in a 1.7 ml microcentrifuge tube at room temperature.

### Cell metabolism

To determine changes in chondrocyte metabolic activity, the cells from each individual sphere were immediately resuspended in 1 ml complete DMEM media containing the tetrazolium MTT reagent (3-(4,5-dimethylthiazol-2-yl)-2,5-diphenyltetrazolium bromide) and incubated at 37 °C in a cell culture incubator, with 14 replicates from either control or stimulated groups used for this analysis. After 3 hours, a solubilization reagent (4 mM HCl, 0.1% NP40 in isopropanol) was added to dissolve the intracellular formazan product into solution, and 100 μl samples of the colored lysate solution was spectrophotometrically analyzed using a plate reader at 590 nm. Statistical analysis was performed using Student’s t-test, n = 14

### Total protein content

Quantification of total protein content of the tissue engineered cartilage was performed using the Bradford assay on 18 replicate samples from each group. In brief, 20 μl of the 1 ml dissociated sample was transferred to wells of a 96-well assay plate, and 100 μl Bradford reagent (Sigma-Aldrich, UK) added, followed by immediate spectrophotometric analysis in a plate reader at 595 nm. Protein in the control and bioreactor samples was quantified against a standard curve of known concentrations of bovine serum albumin (SigmaAldrich, UK). Statistical analysis was performed using Student’s t-test, n = 18

### Glycosaminoglycan content

Quantification of glycosaminoglycan of the tissue engineered cartilage was performed using the dimethylmethylene blue (DMMB) assay. In brief, 20 μl of each 1 ml dissociated sample was transferred to wells of a 96-well assay plate, and 100 μl DMMB reagent was added (16 mg DMMB in 1 litre water containing 3.04 g glycine, 1.6 g NaCl and 95 ml of 0.1 M acetic acid). The samples were analyzed spectrophotometrically in a plate reader at 525 nm. Glycosaminoglycan in the control and bioreactor samples was quantified against a standard curve of known concentrations of bovine trachea chondroitin-4-sulphate (SigmaAldrich, UK). Statistical analysis was performed using Student’s t-test, n = 18

## Results

The motor and driver module were selected based on calculations that the motor must be able to withstand up to 9.93 N m of torque (Table 1). Servo motors offered the required properties, but due to their relatively high cost, other solutions were investigated.^[^^22^^]^ A brushless, closed-loop electric bipolar stepper motor was chosen which combined accurately controlled speed and positioning. Bipolar wiring of the stepper motor offered greater torque due to the single larger coil per winding than the unipolar wiring, which allowed for a stronger magnetic field to be generated, hence greater torque.^[^^23^^]^ The closed-loop stepper motor included in-built feedback from an encoder to the driver which in turn reduced the chance of ‘dropping’ steps under loading, and helped to increase running efficiency, therefore reducing the temperature increase of the motor whilst active.

The crankshaft system was a satisfactory design to produce the required linear displacement (Figure 1F). It was however seen that even slight misalignment of the pneumatic cylinder would result in undesirable movements as the system would attempt to pull the piston further out of the cylinder than was actually possible. For this reason, it was necessary to frequently ensure that cylinder was in the correct location for smoothest operation, which was made possible by the adjustable attachment of the cylinder to the system chassis frame. When correctly aligned, the system ran smoothly, and as expected, pulses of air were output from the cylinder at user-defined amplitudes and frequencies. The method of calibration was found to be too inconsistent to be a permanent solution for the project. Whilst the ultrasonic sensor would work successfully on the majority of tests, on occasion it would be affected by outside noise and would result in failed calibration. The addition of 1-degree alterations performed by the user through the interface aim to counteract this issue, but a more robust and permanent solution is to be investigated.

To test the system prior to use, a 10 A 30 V variable power supply was used which allowed the current drawn from the motor to be monitored in real time and was an aid in understanding the operation of the motor in terms of current draw. Upon completion of the system, this power supply was replaced with a 150 W, 36 V, 4.16 A desktop power supply which allowed the system to be more compact, portable, and suitable for a cell culture laboratory environment. A change in power supply from the original 30 V adjustable power supply to the 150 W 36 V desktop power supply resulted in noticeable improvement in system stability at higher frequencies and amplitudes. Under the use of the previous supply, the system began to appear unstable at frequencies above 3 Hz and at displacements of around 50%. Attempting to drive the system to higher speeds resulted in the system becoming loud in operation and generated positional errors. Using a power supply capable of a higher voltage allowed the system to run more smoothly at 3 Hz and to maximum displacement, with indications that a 48 V power supply would further improve performance, albeit at somewhat greater cost. It was also seen that at greater speeds the resolution of the GUI display graph became poor due to the limit of sending data at 50 ms intervals, such that at faster speeds the data transmission begins to fail. This may be overcome by increasing the baud rate of the serial communication. The graphical user interface was seen to be a valuable addition to the system and the use of C# provided all the necessary classes to achieve what was required. Non-technical test subjects reported that the interface was clear, simple to follow and the operations program was simple to execute.

**Figure 4.**
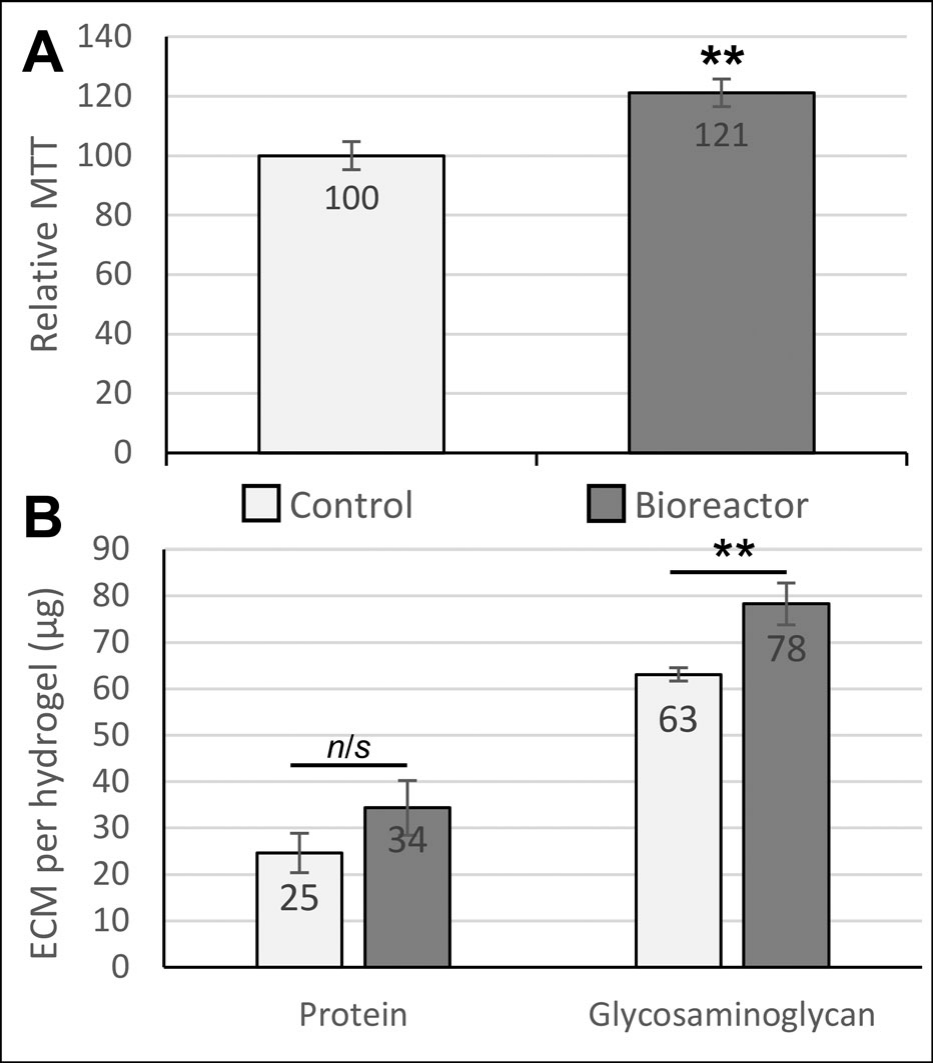
Biological effects of dynamic pressure on chondrocytes in alginate-based hydrogels. (**A**) Chondrocyte proliferation/metabolic activity (measured by MTT assay) was increased by 21% after 5 days of loading in the bioreactor. (**B**) Extracellular matrix (ECM) composition analysis. Both total protein and glycosaminoglycan production by chondrocytes were quantified as biomarkers of cartilage growth. The glycosaminoglycan content in the engineered tissues was significantly increased in response to pulsed pressure. Statistical analysis was performed using Student’s t-test (n = 14-18). ** indicates p < 0.01.

The 3 D printed cell culture chamber (Figure 3) was shown to operate effectively without air leaks or loss of pressure and maintained a sterile internal environment for at least 5 days. Biological testing of the bioreactor was performed by culturing human chondrocytes in a 3 D hydrogel matrix. After 5 days in culture, exposure to pulsed compression in the bioreactor caused an increase in cell metabolic activity of 21% (*p* = 0.008), resulting either from cell proliferation and/or increased metabolic activity per cell (Figure 4A). Quantification of extracellular matrix components revealed that total protein was increased, but not significantly (1.36-fold, *p* = 0.184), and glycosaminoglycan content was significantly increased (1.24-fold increase, *p* = 0.003) compared to parallel controls cultured only in an incubator (Figure 4B).

## Discussion

In this project, our objective was to develop a pulsed pressure bioreactor using affordable commercially available parts, opensource software, and 3 D printing. Our aims were to use this Open Design philosophy to create a bioreactor platform that could be reproduced by other labs, or easily adapted in principal to scale-up, scale-down, or scale-out applications. The core design elements are modular (motor, piston, and a separate cell culture chamber), allowing researchers to upgrade or exchange these elements to suit different pressure regimes. This approach aligns with the growing Open Design Movement for bioinstrumentation, and the increasing interest of researchers to adopt bespoke redesigns of their experimental hardware by using benchtop additive manufacturing tools. In this example, the bioreactor apparatus was co-created by engineers and the end user to better suit the complex and changing demands of experimental workflow.^[^^24^^]^

Our bioreactor design employed a hydropneumatic methodology in which pulsed compressed air was used to generate hydrostatic forces in cell culture media, simulating the type of interstitial fluid compression found in synovial joints and articular cartilage.^[^^15^^,^^25^^]^ A similar approach has previously been used by other researchers and commercial bioreactor manufacturers to generate large pressures in the MPa range by Wenger et al ^[^^26^^]^, and in the kPa range in the TissueGrowth Technologies/Instron/BISS ‘CartiGen HP’ bioreactor.^[^^17^^,^^18^^]^ The CartiGen HP bioreactor is a hydrostatic mechanical compression system running on compressor-pressurized air, designed to impart compressive hydrostatic stress to tissue engineered cartilage constructs in standard polystyrene cell culture plates housed within an aluminum pressure chamber.^[^^17^^]^ The system may achieve a maximum chamber pressure of 300 kPa (3 bar) at frequencies up to 4 Hz, or as limited by the available compressed air supply. An alternative methodology for generating pulsatile hydrostatic forces in culture uses a fully fluid-filled culture chamber which is compressed via an actuator platen acting on a silicon membrane, therefore applying hydrostatic pressure directly to the liquid volume.^[^^27^^]^ This type of bioreactor is exemplified by the versatile TC-3 developed by EBERS. The TC-3 system allows for hydrostatic pressure conditions to be investigated (along with a variety of other mechanical loading profiles) up to a maximum cell culture chamber pressure of 4 bar.

In our practical experience, these systems each have limitations. The large volume of compressed air required by the Cartigen HP bioreactor is hard to cycle whilst maintaining temperature, humidity, and (if needed) a non-atmospheric 5% CO_2_ level to buffer the pH of the culture media. The closed hydrostatic/hydraulic systems using platens and deformable membranes require that all compressible air pockets be eliminated from the compressed volume, and any deficiencies in the sealing (e.g., imperfect gaskets, filling ports) cause immediate ejection of pressurized culture media from weak spots. In our current system, we addressed both of these issues: the entire assembly can be operated in a cell culture incubator, enabling control of gas composition and temperature, whilst the use of compressed air rather than compressed liquid means that leaks are easier to detect and resolve without catastrophic failure and compromised sterility. Our approach of using compressed air, rather than compressed liquid means that leaks in the sealing are less catastrophic (i.e., pressurized cell culture media is not sprayed throughout the incubator). Our inclusion of pressure sensors to continuously check for leaks has helped track and improve the sealing strategy, which is now optimized in this O-ring and bolt pattern arrangement.

PLA has proved to be a reliable material for producing functional pressurized prototypes when print settings are optimized to minimize layer distortion and at 100% in-fill. We have previously trialed several other polymers for 3 D printing bioreactors including ABS, nylon (which is hygroscopic and tends to distort), and a variety of dental resins, which proved to be brittle post curing. In our previous cell culture experiments, PLA-based bioreactor chambers have shown no cytotoxicity after repeat washes with saline solution (ISO 10993 testing), are effectively sterilized using 70% ethanol/water, and can be coated with clear polydimethylsiloxane PDMS) resin for improved durability.^[^^14^^]^

Whilst the development of the bioreactor was successful, we have identified areas for improvement in subsequent designs. Our observations whilst testing the bioreactor were that changes in power supply from the original 30 V adjustable power supply to the 150 W 36 V desktop power supply improved the stability of the system at higher frequencies, but 4 Hz was still a practical upper limit beyond which rapid reciprocation of the linkage became problematic. It is likely that a more refined design of linkage, piston dampers, and improved programming the motor to accelerate and decelerate briefly into each pulse would improve the smoothness of operation, together with a higher voltage 48 V power supply.^[^^23^^]^ It was also seen that at greater speeds, the resolution of the graph displayed became poor due to the limit of sending data at 50 ms intervals, and so this can probably be overcome by increasing the baud rate of the serial communication.^[^^28^^]^ Overall, the performance of the bioreactor at the end of the engineering development phase was satisfactory, and the convenience of the user interface enabled scientists without programming or engineering experience to safely and reproducibly use the device to successfully culture cells.

Cell culture studies were performed to validate the effectiveness of the bioreactor in eliciting responses from mechanosensitive chondrocytes when cultured in a 3 D ‘tissue like’ hydrogel, a widely used method for obtaining physiologically relevant data from these cells *in vitro*.^[^^6^^,^^9^^,^^21^^]^ The increase in cell metabolism as measured by MTT resulted either from increased cell proliferation and/or increased metabolic activity per cell, both of which have previously been reported as outcomes from mechanically stimulated chondrocytes.^[^^12^^,^^29–31^^]^ Similarly, our observations of increases in glycosaminoglycans (a key biomarker of cartilage extracellular matrix) are consistent with the literature, and further demonstrate the effectiveness of this bioreactor in generating mechanical stimuli that are detectable by chondrocytes.^[^^19^^,^^32^^]^

The maximum pressure reliably achieved in this design was 300 kPa, which was sufficient for our experimental applications but an order of magnitude lower than some other direct hydrostatic compression bioreactors used for research in cartilage tissue engineering and mechanobiology, which can range up to 10 MPa^[^^26^^,^^33^^]^. Whilst it is probable that the general design can be extended past this range with more robust materials, our original proposal was to produce a bioreactor capable of exploring the effects on cells of lower ranges of pressure and higher ranges of frequency.^[^^17^^]^ Our objectives for ongoing research are to use this bioreactor platform for further studies into the effects of constitutive microenvironmental forces on cartilage and engineered tissues.

## Supplementary Material

Supplemental Material
