# Supplementary material for: Pneumatic piston hydrostatic bioreactor for cartilage tissue engineering
Source: Instrum Sci Technol. 2022 Sep 20;51(3):273–89. doi: 10.1080/10739149.2022.2124418 (PMC10041975; doi:10.1080/10739149.2022.2124418)
Supplement: Supplemental Material [file LIST_A_2124418_SM2166.docx]

**SUPPLEMENTARY MATERIAL**

**Pneumatic piston hydrostatic bioreactor for cartilage tissue engineering**

**J. Hallas^1,2,3^, A. J. Janvier^1,2^, K. F. Hoettges^3^, J. R. Henstock^1,2^***

1. Department of Musculoskeletal and Ageing Science, Institute of Life Course and Medical Sciences, University of Liverpool, Liverpool L7 8TX, UK
2. The Medical Research Council Versus Arthritis Centre for Integrated Research into Musculoskeletal Ageing (CIMA)
3. Department of Electrical Engineering and Electronics, University of Liverpool, Liverpool L69 3GJ, UK

Corresponding author: Dr James R Henstock

Mailing address: Department of Musculoskeletal and Ageing Science

Institute of Life Course and Medical Sciences

University of Liverpool, Liverpool L7 8TX, UK

Telephone: +61 433 801 372 / +44 7793 886 098

Email: [j.r.henstock@liverpool.ac.uk](mailto:j.r.henstock@liverpool.ac.uk)

**Table S1.** Component costs for the bioreactor, 2022 (UK).

| **Component** | **Cost** |
| --- | --- |
| Motor | £93.43 |
| Driver | £50.44 |
| Pneumatic cylinder | £254.36 |
| Arduino Uno | £26.00 |
| Aluminium/machining costs | £100 |
| 3D printing | £10 |
| **Total** | **£534.23** |

**Table S2.** CL86T Driver Pin Definitions.

| **Pin** | **Input/Output** | **Description** |
| --- | --- | --- |
| Pul+ | Input | Pulse signal: switching this pin from high to low provides movement of a single step. The time between each step can be configured to result in different speeds (depending on step size). |
| Dir+ | Input | Direction signal: in the configuration shown, setting this pin high results in clockwise movement and low to counter clockwise. |
| Ena+ | Input | Enable signal: in the configuration shown, setting this pin high disables the motor (will not power the motor coils) and setting low powers the motor. |
| Pend+ | Output | Encoder position error signal: pin is high when the difference between actual position and intended position is zero and thus is in the correct position. |
| Alm+ | Output | Alarm signal: pin is high when any of the following protection mechanisms have been activated; over-voltage, over current, or position following error. |

**Fig. S1.** Complete wiring schematic for the bioreactor assembly.

**Appendix A: Engineering calculations**

**A1: Pneumatic cylinder volume**

𝑉= 𝜋𝑟^2^ℎ= 𝜋∙(25)^2^∙80 = 157079.6 𝑚𝑚^3^ ≈ 157 𝑚𝑙

**A2: Pneumatic cylinder volume**

At a pressure of 4 bar,

4 = 157+𝑉_𝑆𝑃𝐴𝑅𝐸_ ∴ 𝑉_𝑆𝑃𝐴𝑅𝐸_ = 157 = 52.3̇

𝑉_𝑆𝑃𝐴𝑅𝐸_ (4-1)

**A3: Approximation of crankshaft equation**

Beginning with the equation shown below,


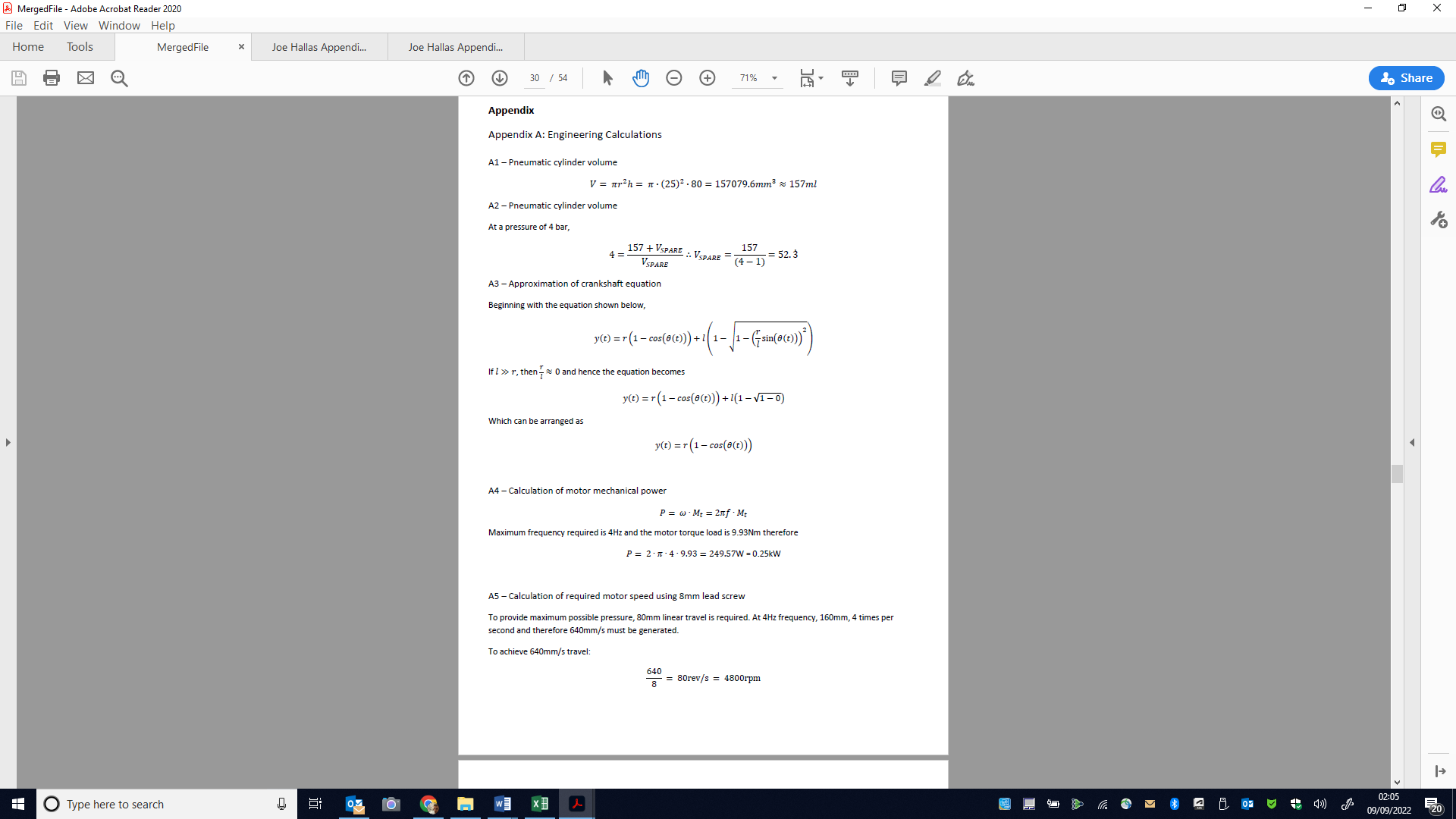


If 𝑙≫𝑟, then 𝑟𝑙 ≈ 0 and hence the equation becomes

𝑦(𝑡)=𝑟(1−𝑐𝑜𝑠(𝜃(𝑡)))+𝑙(1−√1−0)

Which can be arranged as

𝑦(𝑡)=𝑟(1−𝑐𝑜𝑠(𝜃(𝑡)))

**A4: Calculation of motor mechanical power**

𝑃 = 𝜔∙𝑀𝑡 = 2𝜋𝑓∙𝑀𝑡

The maximum frequency required is 4 Hz and the motor torque load is 9.93 N m, therefore

𝑃 = 2∙𝜋∙4∙9.93 = 249.57W = 0.25kW

**A5: Calculation of required motor speed using 8mm lead screw**

To provide maximum possible pressure, 80 mm linear travel is required. At 4 Hz frequency, 160 mm, 4 times per second and therefore 640 mm/s must be generated.

To achieve 640 mm/s travel:

640 = 80 rev/s = 4800 rpm

8

**Appendix C1: MATLAB code for force calculation**

r = 4; %%radius in cm

i = 1;

stepdeg = 1.8;

x = zeros((360/stepdeg), 6, 'single'); %%pre-allocation of results matrix

disp = 8; %%maximum displacement of piston

cylvol = (disp)*pi*(2.5^2); %%cyclinder volume in ml

nullvol = 53.3; %%spare volume in wells/tubing

initvol = cylvol+nullvol;%%initial volume

pistonSA = pi*(0.025^2);%%piston surface area

for theta = 0:stepdeg:360 %%Calculates linear displacement over full rotation

x(i,1) = r*(1-cosd(theta)); %%linear displacement

x(i,2) = theta; %%angular displacement

x(i,3) = (initvol)/( ( 8-x(i,1) )*pi*(2.5^2) + nullvol); %%Pressure in BAR

x(i,4) = ((x(i,3)-1))*pistonSA*10^5; %%Force returning to piston in Newtons

x(i,5) = r*sind(theta); %%vertical distance

x(i,6) = abs((x(i,5)/100) * x(i,4)); %%moment acting on shaft

i=i+1;

end

[maxTorque, rowIdxtorque] = max(x(:,6),[],1);%%maximum torque and location

[maxForce, rowIdx] = max(x(:,4),[],1);%%maximum force and location

fprintf('Maximum torque: %.2fNm\n', maxTorque);

fprintf('Experienced at: %.1fDegrees', x(rowIdxtorque,2));

fprintf('\nMaximum force received: %.2fN', maxForce);

fprintf('\nAchieved pressure: %.2fBAR\n', max(x(:,3)));

%%Graphing Controls

plot(x(:,2), x(:,6), 'b', 'LineWidth',1);

hold on;

plot(x(:,2), x(:,3), 'r', 'LineWidth',1);

title({'Chamber pressure and moment acting on motor shaft through a full rotation', '(assuming 73ml empty volume in tubing/chamber)'})

xlabel('Angular Displacement (°)')

ylabel('Moment(Nm) /Chamber Pressure (BAR)')

legend('Moment acting on shaft','Pressure in chamber')

grid on

grid minor

xlim([0 360])

xticks([0 30 60 90 120 150 180 210 240 270 300 330 360])

**Appendix C2: ARDUINO code for bioreactor operation**

#include <Timer.h>//library to include timed interrupts

int trig = 3;//sr04

int echo = 4;//sr04

int pul = 9; //pulse+ pin

int ena = 7; //enable+ pin

int dir = 8; //dir+ pin

int alarm = 5; //alarm+ pin

int pend = 6; //pend+ pin

int pos;//variable to store current motor position in num. steps

bool senddata;//flag to decide whether to transmit motor position to serial

Timer t;//declares a timer

void setup() {

pinMode(13, OUTPUT);

pinMode(A0, INPUT);

pinMode(pul, OUTPUT);

pinMode(ena, OUTPUT);

pinMode(dir, OUTPUT);

pinMode(pend, INPUT);

pinMode(alarm, INPUT);

pinMode(echo, INPUT);

pinMode(trig, OUTPUT);

digitalWrite(ena, HIGH); //Motor off by defualt

Serial.begin(9600);

senddata = false;//do not send motor position by default

pos = 0;//position begins at 0

}

void loop() {

if (Serial.available())

{

delay(5);//necessary delay to ensure messages are picked up

String data = Serial.readStringUntil(':');//read data from serial port until a ':' is read

if (data == "c")

{

calibrate();

}

else if (data == "cw")

{

digitalWrite(ena, LOW);//activates motor

delayMicroseconds(5);

digitalWrite(dir, HIGH);//clockwise direction

delayMicroseconds(5);

for (int m = 0; m < 3; m++)//move 3 steps (1 degree) clockwise

{

digitalWrite(pul, LOW);

delayMicroseconds(1000);

digitalWrite(pul, HIGH);

delayMicroseconds(100);

}

}

else if (data == "acw")

{

digitalWrite(ena, LOW);//activates motor

delayMicroseconds(5);

digitalWrite(dir, LOW);//anticlockwise direction

delayMicroseconds(5);

for (int m = 0; m < 3; m++)//move 3 steps (1 degree) anticlockwise

{

digitalWrite(pul, LOW);

delayMicroseconds(1000);

digitalWrite(pul, HIGH);

delayMicroseconds(100);

}

}

else if (isDigit(data.charAt(0)))//if data read until ':' is a number

{

int num_steps = data.toInt();//convert data into an integer

String str = Serial.readStringUntil(':');//read serial until next ':' recieved

double usec_p_step = str.toFloat();//convert new data to float and store

str = Serial.readStringUntil(':');//read serial until next ':' recieved

int num_pulses = str.toInt();//convert new data to integer and store

senddata = true;//send motor position to serial

digitalWrite(ena, LOW);//activate motor

delayMicroseconds(10);//delay necessary to activate motor

run_program(num_steps, usec_p_step, num_pulses);//send data read to function

}

}

}

void run_program(int num_steps, double usec_p_step, int num_pulses)

{

t.every(50, takeReading);//every 50ms send motor data

for (int j = 0; j < num_pulses; j++)

{

{

delayMicroseconds(5);

digitalWrite(dir, HIGH);//clockwise

delayMicroseconds(5);

for (pos = 0; pos < num_steps; pos++)//move to maximum

{

t.update();

digitalWrite(pul, LOW);

delayMicroseconds(usec_p_step);

digitalWrite(pul, HIGH);

delayMicroseconds(10);

}

delayMicroseconds(5);

digitalWrite(dir, LOW);//anticlockwise

delayMicroseconds(5);

for (pos = num_steps; pos > 0; pos--)//return to 0 position

{

t.update();//timer requires updating to ensure successful interrupts

digitalWrite(pul, LOW);

delayMicroseconds(usec_p_step);

digitalWrite(pul, HIGH);

delayMicroseconds(10);

}

}

if (Serial.available())//if data is available to be read

{

String data = Serial.readStringUntil(':');

if (data == "s")//signifies a 'stop' instruction

{

break;//exit program

}

}

}

Serial.print("done\n");//lets the UI know the program is complete

}

void calibrate()

{

digitalWrite(ena, LOW);

delayMicroseconds(5);

digitalWrite(dir, LOW);

delayMicroseconds(5);

while (SR04Distance() > 8)//while system is not at max displacement

{//move another step

digitalWrite(pul, LOW);

delayMicroseconds(1000);

digitalWrite(pul, HIGH);

delayMicroseconds(100);

}

String tosend = "c" + String(3.57) + "\n";

Serial.print(tosend);

delayMicroseconds(5);

digitalWrite(dir, HIGH);

delayMicroseconds(5);

**Appendix C3: C# code for user interface**

using System;

using System.Collections.Generic;

using System.ComponentModel;

using System.Data;

using System.Drawing;

using System.Linq;

using System.Text;

using System.Threading.Tasks;

using System.Windows.Forms;

using System.IO.Ports;

using System.Threading;

using ZedGraph;//used for graphing motor position

namespace BioreactorGUI

{

public partial class Title : Form

{

private System.IO.Ports.SerialPort COMport;

GraphPane panepressure = new GraphPane();//creates new graph

PointPairList listpointspressure = new PointPairList();//list of points to store graph values

LineItem pressurecurve;//creates the actual graph curve

int n_readings = 0;//initialises to number of motor locations recieved to 0

double max_pressure;//creates a variable to store the max pressure which will be returned from Arduino upon calibration

public Title()

{

InitializeComponent();

//prepares for use by disabling the necessary functions

Calibrate_button.Enabled = false;

Stop_button.Enabled = false;

Start_button.Enabled = false;

Pressure_box.ReadOnly = true;

Max_pressure_box.Visible = false;

Error_icon.Visible = true;

Ready_icon.Visible = true;

Anticlockwise.Enabled = false;

Clockwise.Enabled = false;

Port_selection_box.Items.AddRange(SerialPort.GetPortNames());//populates serial port selection box

if (Port_selection_box.Items.Count < 1)

{

Log_box.Text += "No serial connections were found" + "\r\n";

Ready_icon.Visible = false;//makes error icon visible

}

else

{

Log_box.Text += "Serial connections found" + "\r\n";

Ready_icon.Visible = true;

}

}

private void Form1_Load(object sender, EventArgs e)

{

SetupGraph();

}

private void SetupGraph()

{

panepressure = zedGraphControl1.GraphPane;

panepressure.Title.Text = "Pressure";

panepressure.XAxis.Title.Text = "Time (s)";

panepressure.YAxis.Title.Text = "Pressure (BAR)";

panepressure.YAxis.Scale.MaxAuto = true;

panepressure.YAxis.Scale.MinAuto = true;

panepressure.XAxis.Scale.MaxAuto = true;

panepressure.XAxis.Scale.MinAuto = true;

zedGraphControl1.IsZoomOnMouseCenter = true;

pressurecurve = panepressure.AddCurve(null, listpointspressure, Color.Black, SymbolType.None);

pressurecurve.Line.Width = 3;

}

private void Title_label_Click(object sender, EventArgs e)

{

}

private void panel1_Paint(object sender, EventArgs e)

{

}

private void Calibrate_button_Click(object sender, EventArgs e)

{

string Port_Name = Port_selection_box.SelectedItem.ToString();

if(COMport == null)

{

COMport = new SerialPort(Port_Name, 9600, Parity.None, 8, StopBits.One);

}

COMport.DataReceived += new SerialDataReceivedEventHandler(Port_OnDataReceived);//creates an event handler for when serial data is recieved

if (!COMport.IsOpen)

{

try

{

COMport.Open();

}

#region

catch (UnauthorizedAccessException SerialException) //exception thrown when the operating system denies access

{

MessageBox.Show(SerialException.ToString());

Log_box.Text = Port_Name + "\r\n";

Log_box.Text += SerialException.ToString() + "\r\n";

Ready_icon.Visible = false;

COMport.Close();

}

catch (System.IO.IOException SerialException) //An attempt to set the state of the port failed. I.e. the port is in an invalid state

{

MessageBox.Show(SerialException.ToString());

Log_box.Text = Port_Name + "\r\n";

Log_box.Text += SerialException.ToString() + "\r\n";

Ready_icon.Visible = false;

COMport.Close();

}

catch (InvalidOperationException SerialException) //The specified port on the current SerialPort object is already open

{

MessageBox.Show(SerialException.ToString());

Log_box.Text = Port_Name + "\r\n";

Log_box.Text += SerialException.ToString() + "\r\n";

Ready_icon.Visible = false;

COMport.Close();

}

catch //Any other errors

{

MessageBox.Show("Error in opening serial port - unknown error");

Ready_icon.Visible = false;

COMport.Close();

}

#endregion

}

if (COMport.IsOpen == true) //if the port was successfully opened

{

COMport.Write("c:");

Log_box.Text += "Calibrating system - please wait" + "\r\n";

Thread.Sleep(10);

Calibrate_button.Enabled = false;//prevents resending calibration instruction to Arduino

}

else

{

MessageBox.Show("Unable to communicate with serial port");

COMport.Close();

}

}

private void Start_button_Click(object sender, EventArgs e)

{

listpointspressure.Clear();//prepares a new list of values for the graph

panepressure.AxisChange();//resets axis

zedGraphControl1.Refresh();//prepares new graph

zedGraphControl1.ZoomOutAll(panepressure);

string Port_Name = Port_selection_box.SelectedItem.ToString();//gets port name from selection box

if (COMport == null)

{

COMport = new SerialPort(Port_Name, 9600, Parity.None, 8, StopBits.One);

}

COMport.DataReceived += new SerialDataReceivedEventHandler(Port_OnDataReceived);//creates event handler for new serial port

if (!COMport.IsOpen)

{

try

{

COMport.Open();

}

#region

catch (UnauthorizedAccessException SerialException) //exception thrown when the operating system denies access

{

MessageBox.Show(SerialException.ToString());

Log_box.Text = Port_Name + "\r\n";

Log_box.Text += SerialException.ToString() + "\r\n";

Ready_icon.Visible = false;

COMport.Close();

}

catch (System.IO.IOException SerialException) //An attempt to set the state of the port failed. I.e. the port is in an invalid state

{

MessageBox.Show(SerialException.ToString());

Log_box.Text = Port_Name + "\r\n";

Log_box.Text += SerialException.ToString() + "\r\n";

Ready_icon.Visible = false;

COMport.Close();

}

catch (InvalidOperationException SerialException) //The specified port on the current SerialPort object is already open

{

MessageBox.Show(SerialException.ToString());

Log_box.Text = Port_Name + "\r\n";

Log_box.Text += SerialException.ToString() + "\r\n";

Ready_icon.Visible = false;

COMport.Close();

}

catch //Any other error

{

MessageBox.Show("Error in opening serial port - unknown error");

Ready_icon.Visible = false;

COMport.Close();

}

#endregion

}

if (COMport.IsOpen == true)

{

if((Frequency_box.Text == "") || (Num_pulses_box.Text == "") || (Pressure_box.Text == ""))

{//if program data is not complete

MessageBox.Show("Please enter values into all program parameters in order to start program.", "Error", MessageBoxButtons.OK, MessageBoxIcon.Error);

}

if (float.TryParse(Frequency_box.Text, out float frequency))

if (int.TryParse(Num_pulses_box.Text, out int num_pulses))

if (float.TryParse(Pressure_box.Text, out float pressure))

if ((frequency != 0)&&(num_pulses!=0)&&(pressure!=0))

{//if all user entered values have been succesfully parsed to values

double lin_disp = (pressure / max_pressure) * 80;//linear displacement calculation

double angle = (Math.Acos(1 - (lin_disp / 40)))*57.29577951;//Calculates required angle to achieve displacement

int num_steps = (int)((angle / 0.36) + 0.5);//calculates number of steps to achieve angle

double tpstep = (((1 / frequency) - 0.00002) / 2)/num_steps;//time per step in seconds

double usecspstep = ((tpstep * 1000000) - 10);//time per step in microseconds

string tosend = num_steps.ToString() + ":" + usecspstep.ToString("F2") + ":" + num_pulses.ToString() + ":";

Log_box.Text += tosend + "\r\n";

COMport.Write(tosend);

Log_box.Text += "Sending program to controller" + "\r\n";

Log_box.Text += "Frequency: " + frequency + "Hz" + ", ";

Log_box.Text += "Pressure: " + pressure + " BAR, ";

Log_box.Text += "For: " + num_pulses + " pulses" + "\r\n";

Thread.Sleep(10);

Start_button.Enabled = false;

Stop_button.Enabled = true;

n_readings = 0;

}

}

else

{

MessageBox.Show("Unable to communicate with serial port");

COMport.Close();

}

}

private void Setup_title_Click(object sender, EventArgs e)

{

}

private void Port_selection_label_Click(object sender, EventArgs e)

{

}

private void Port_selection_box_SelectedIndexChanged(object sender, EventArgs e)

{

string Selected_Port = Port_selection_box.SelectedItem.ToString() + " Selected"; // Store the Selected COM port

Log_box.Text += Selected_Port + "\r\n";

Calibrate_button.Enabled = true;

Anticlockwise.Enabled = true;

Clockwise.Enabled = true;

SerialPort COMport = new SerialPort(Selected_Port, 9600, Parity.None, 8, StopBits.One);

COMport.DataReceived += new SerialDataReceivedEventHandler(Port_OnDataReceived);

}

private void Port_OnDataReceived(object sender, SerialDataReceivedEventArgs e)

{

SerialPort sp = (SerialPort)sender;

string reply = sp.ReadLine();

if (reply[0] == 'c')

{

string n_reply = reply.Remove(0,1);//removes first character which will be 'c'

this.Invoke(new MethodInvoker(delegate ()

{

Log_box.Text += "Calibration successful. System is ready." + "\r\n";

Stop_button.Enabled = true;

Start_button.Enabled = true;

Ready_icon.Visible = true;

Pressure_box.ReadOnly = false;

if(float.TryParse(n_reply, out float cal_press))//parse remaining data (max pressure) to a float

max_pressure = cal_press;

Max_pressure_box.Visible = true;

Max_pressure_box.Text = "(Max: " + (max_pressure.ToString("n2")) + ")\r\n";

Thread.Sleep(10);

Calibrate_button.Enabled = true;

}));

}

else if (Char.IsDigit(reply[0]) == true)

{

this.Invoke(new MethodInvoker(delegate ()

{

n_readings++;

double numsteps = double.Parse(reply);

double degrees = numsteps * 0.36;

double lin_disp = 40 * (1 - Math.Cos(degrees*0.01745329));

double pressure = (lin_disp / 80) * max_pressure;

listpointspressure.Add(new PointPair(((double)n_readings / 20), pressure));

panepressure.AxisChange();

zedGraphControl1.Refresh();

}));

}

switch (reply)

{

case "poserr":

{

this.Invoke(new MethodInvoker(delegate ()

{

Log_box.Text += "Encoder position error" + "\r\n";

Ready_icon.Visible = false;

Thread.Sleep(10);

}));

}

break;

case "alarm":

{

this.Invoke(new MethodInvoker(delegate ()

{

Log_box.Text += "Over current/voltage alarm. Action stopped" + "\r\n";

Ready_icon.Visible = false;

Thread.Sleep(10);

}));

}

break;

case "done":

{

this.Invoke(new MethodInvoker(delegate ()

{

Log_box.Text += "Program complete." + "\r\n";

Ready_icon.Visible = true;

Start_button.Enabled = true;

Stop_button.Enabled = false;

Thread.Sleep(10);

}));

}break;

}

}

private void Control_title_Click(object sender, EventArgs e)

{

}

private void Stop_button_Click(object sender, EventArgs e)

{

string Port_Name = Port_selection_box.SelectedItem.ToString();

if (COMport == null)

{

COMport = new SerialPort(Port_Name, 9600, Parity.None, 8, StopBits.One);

}

COMport.DataReceived += new SerialDataReceivedEventHandler(Port_OnDataReceived);

if (!COMport.IsOpen)

{

try

{

COMport.Open();

}

#region

catch (UnauthorizedAccessException SerialException) //exception thrown when the operating system denies access

{

MessageBox.Show(SerialException.ToString());

Log_box.Text = Port_Name + "\r\n";

Log_box.Text += SerialException.ToString() + "\r\n";

Ready_icon.Visible = false;

COMport.Close();

}

catch (System.IO.IOException SerialException)//An attempt to set the state of the port failed. I.e. the port is in an invalid state

{

MessageBox.Show(SerialException.ToString());

Log_box.Text = Port_Name + "\r\n";

Log_box.Text += SerialException.ToString() + "\r\n";

Ready_icon.Visible = false;

COMport.Close();

}

catch (InvalidOperationException SerialException) //The specified port on the current SerialPort object is already open

{

MessageBox.Show(SerialException.ToString());

Log_box.Text = Port_Name + "\r\n";

Log_box.Text += SerialException.ToString() + "\r\n";

Ready_icon.Visible = false;

COMport.Close();

}

catch //Any other ERROR

{

MessageBox.Show("Error in opening serial port - unknown error");

Ready_icon.Visible = false;

COMport.Close();

}

#endregion

}

if (COMport.IsOpen == true)

{

COMport.Write("s:");

Thread.Sleep(10);

Start_button.Enabled = false;

Stop_button.Enabled = false;

Calibrate_button.Enabled = true;

Log_box.Text += "Program stopped." + "\r\n";

}

else

{

MessageBox.Show("Unable to communicate with serial port");

COMport.Close();

}

}

private void panel3_Paint(object sender, PaintEventArgs e)

{

}

private void Log_box_TextChanged(object sender, EventArgs e)

{

Log_box.SelectionStart = Log_box.Text.Length;

Log_box.ScrollToCaret();//automatically scroll log box to recent entries

}

private void Refresh_ports_button_Click(object sender, EventArgs e)

{

Port_selection_box.Items.Clear();

Port_selection_box.Items.Remove(Port_selection_box.SelectedItem);

Port_selection_box.Items.AddRange(SerialPort.GetPortNames());

if (Port_selection_box.Items.Count < 1)

{

Log_box.Text += "No serial connections were found" + "\r\n";

Ready_icon.Visible = false;

}

else { Log_box.Text += "Serial connections found" + "\r\n";

Ready_icon.Visible = true;}

}

private void panel6_Paint(object sender, PaintEventArgs e)

{

}

private void label4_Click(object sender, EventArgs e)

{

}

private void pictureBox1_Click(object sender, EventArgs e)

{

}

private void Frequency_box_TextChanged(object sender, EventArgs e)//ensures correct entries are only accepted

{

if (float.TryParse(Frequency_box.Text, out float frequency))

{

if ((frequency > 3.5) || (frequency < 0))

{

Pressure_box.Text = "";

MessageBox.Show("Frequency may not be above 3.5 Hz", "Error", MessageBoxButtons.OK, MessageBoxIcon.Error);

}

}

if (float.TryParse(Frequency_box.Text, out float freq))

if (int.TryParse(Num_pulses_box.Text, out int num))

if (freq != 0 && num != 0)

{

float t_total = (num) / (freq); //total time in seconds

T_total_box.Text = t_total.ToString();

}

}

private void Frequency_box_KeyPress(object sender, KeyPressEventArgs e)

{

char ch = e.KeyChar;//gets entered character

if(!char.IsDigit(ch) && ch !=8 && ch != 46 && ch != '.' && ch != 13)

{

e.Handled = true; //ensures only valid data can be entered

}

if (int.TryParse(Frequency_box.Text, out int result))

if (int.TryParse(Num_pulses_box.Text, out result))

if (float.Parse(Frequency_box.Text) != 0 && float.Parse(Num_pulses_box.Text) != 0)

{

float t_total = (float.Parse(Num_pulses_box.Text)) / (float.Parse(Frequency_box.Text)); //total time in seconds

T_total_box.Text = t_total.ToString();

}

}

private void Title_KeyDown(object sender, KeyEventArgs e)

{//make the 'enter' key act as 'tab' key

if (e.KeyCode == Keys.Enter)

{

SendKeys.Send("{TAB}");

e.Handled = true;

}

}

private void Num_pulses_box_KeyPress(object sender, KeyPressEventArgs e)

{

char ch = e.KeyChar;

if (!char.IsDigit(ch) && ch != 8 && ch != 46 && ch != 13)//if entry in not a digit, or backspace or '.' etc...

{

e.Handled = true; //ensures only valid data can be entered

}

}

private void Num_pulses_box_TextChanged(object sender, EventArgs e)

{

if (float.TryParse(Frequency_box.Text, out float freq))

if (int.TryParse(Num_pulses_box.Text, out int num))

if (freq != 0 && num != 0)

{

float t_total = (num) / (freq); //total time in seconds

TimeSpan t = TimeSpan.FromSeconds(t_total);

string time = t.ToString(@"hh\:mm\:ss");

T_total_box.Text = time;

}

}

private void T_total_box_TextChanged(object sender, EventArgs e)

{

}

private void label5_Click_1(object sender, EventArgs e)

{

}

private void Max_pressure_box_TextChanged(object sender, EventArgs e)

{

}

private void Pressure_box_TextChanged(object sender, EventArgs e)

{

if(float.TryParse(Pressure_box.Text, out float pressure))

{

if ((pressure > max_pressure)||(pressure < 0))//if pressure entered is out of range

{

Pressure_box.Text = "";//clear text box

MessageBox.Show("Pressure must be a positive value below the maximum value shown", "Error", MessageBoxButtons.OK, MessageBoxIcon.Error);

}

}

}

private void Pressure_box_KeyPress(object sender, KeyPressEventArgs e)

{

char ch = e.KeyChar;

if (!char.IsDigit(ch) && ch != 8 && ch != 46 && ch != 13)

{

e.Handled = true; //ensures only valid data can be entered

}

}

private void pictureBox2_Click(object sender, EventArgs e)

{

string Port_Name = Port_selection_box.SelectedItem.ToString();

if (COMport == null)

{

COMport = new SerialPort(Port_Name, 9600, Parity.None, 8, StopBits.One);

}

COMport.DataReceived += new SerialDataReceivedEventHandler(Port_OnDataReceived);

if (!COMport.IsOpen)

{

try

{

COMport.Open();

}

#region

catch (UnauthorizedAccessException SerialException) //exception that is thrown when the operating system denies access

{

MessageBox.Show(SerialException.ToString());

Log_box.Text = Port_Name + "\r\n";

Log_box.Text += SerialException.ToString() + "\r\n";

Ready_icon.Visible = false;

COMport.Close();

}

catch (System.IO.IOException SerialException) // An attempt to set the state of the underlying port failed

{

MessageBox.Show(SerialException.ToString());

Log_box.Text = Port_Name + "\r\n";

Log_box.Text += SerialException.ToString() + "\r\n";

Ready_icon.Visible = false;

COMport.Close();

}

catch (InvalidOperationException SerialException) // The specified port on the current instance of the SerialPort is already open

{

MessageBox.Show(SerialException.ToString());

Log_box.Text = Port_Name + "\r\n";

Log_box.Text += SerialException.ToString() + "\r\n";

Ready_icon.Visible = false;

COMport.Close();

}

catch //Any other ERROR

{

MessageBox.Show("Error in opening serial port - unknown error");

Ready_icon.Visible = false;

COMport.Close();

}

#endregion

}

if (COMport.IsOpen == true)

{

COMport.Write("cw:");

Log_box.Text += "Moving 1 degree clockwise" + "\r\n";

}

else

{

MessageBox.Show("Unable to communicate with serial port");

COMport.Close();

}

}

private void pictureBox3_Click(object sender, EventArgs e)

{

string Port_Name = Port_selection_box.SelectedItem.ToString();

if (COMport == null)

{

COMport = new SerialPort(Port_Name, 9600, Parity.None, 8, StopBits.One);

}

COMport.DataReceived += new SerialDataReceivedEventHandler(Port_OnDataReceived);

if (!COMport.IsOpen)

{

try

{

COMport.Open();

}

#region

catch (UnauthorizedAccessException SerialException) //exception that is thrown when the operating system denies access

{

MessageBox.Show(SerialException.ToString());

Log_box.Text = Port_Name + "\r\n";

Log_box.Text += SerialException.ToString() + "\r\n";

Ready_icon.Visible = false;

COMport.Close();

}

catch (System.IO.IOException SerialException) // An attempt to set the state of the underlying port failed

{

MessageBox.Show(SerialException.ToString());

Log_box.Text = Port_Name + "\r\n";

Log_box.Text += SerialException.ToString() + "\r\n";

Ready_icon.Visible = false;

COMport.Close();

}

catch (InvalidOperationException SerialException) // The specified port on the current instance of the SerialPort is already open

{

MessageBox.Show(SerialException.ToString());

Log_box.Text = Port_Name + "\r\n";

Log_box.Text += SerialException.ToString() + "\r\n";

Ready_icon.Visible = false;

COMport.Close();

}

catch //Any other ERROR

{

MessageBox.Show("Error in opening serial port - unknown error");

Ready_icon.Visible = false;

COMport.Close();

}

#endregion

}

if (COMport.IsOpen == true)

{

COMport.Write("acw:");

Log_box.Text += "Moving 1 degree anticlockwise" + "\r\n";

}

else

{

MessageBox.Show("Unable to communicate with serial port");

COMport.Close();

}

}

private void Clockwise_Click(object sender, EventArgs e)

{

string Port_Name = Port_selection_box.SelectedItem.ToString();

if (COMport == null)

{

COMport = new SerialPort(Port_Name, 9600, Parity.None, 8, StopBits.One);

}

COMport.DataReceived += new SerialDataReceivedEventHandler(Port_OnDataReceived);

if (!COMport.IsOpen)

{

try

{

COMport.Open();

}

#region

catch (UnauthorizedAccessException SerialException) //exception that is thrown when the operating system denies access

{

MessageBox.Show(SerialException.ToString());

Log_box.Text = Port_Name + "\r\n";

Log_box.Text += SerialException.ToString() + "\r\n";

Ready_icon.Visible = false;

COMport.Close();

}

catch (System.IO.IOException SerialException) // An attempt to set the state of the underlying port failed

{

MessageBox.Show(SerialException.ToString());

Log_box.Text = Port_Name + "\r\n";

Log_box.Text += SerialException.ToString() + "\r\n";

Ready_icon.Visible = false;

COMport.Close();

}

catch (InvalidOperationException SerialException) // The specified port on the current instance of the SerialPort is already open

{

MessageBox.Show(SerialException.ToString());

Log_box.Text = Port_Name + "\r\n";

Log_box.Text += SerialException.ToString() + "\r\n";

Ready_icon.Visible = false;

COMport.Close();

}

catch //Any other ERROR

{

MessageBox.Show("Error in opening serial port - unknown error");

Ready_icon.Visible = false;

COMport.Close();

}

#endregion

}

if (COMport.IsOpen == true)

{

COMport.Write("cw:");

Log_box.Text += "Moving 1 degree clockwise" + "\r\n";

}

else

{

MessageBox.Show("Unable to communicate with serial port");

COMport.Close();

}

}

private void Anticlockwise_Click(object sender, EventArgs e)

{

string Port_Name = Port_selection_box.SelectedItem.ToString();

if (COMport == null)

{

COMport = new SerialPort(Port_Name, 9600, Parity.None, 8, StopBits.One);

}

COMport.DataReceived += new SerialDataReceivedEventHandler(Port_OnDataReceived);

if (!COMport.IsOpen)

{

try

{

COMport.Open();

}

#region

catch (UnauthorizedAccessException SerialException) //exception that is thrown when the operating system denies access

{

MessageBox.Show(SerialException.ToString());

Log_box.Text = Port_Name + "\r\n";

Log_box.Text += SerialException.ToString() + "\r\n";

Ready_icon.Visible = false;

COMport.Close();

}

catch (System.IO.IOException SerialException) // An attempt to set the state of the underlying port failed

{

MessageBox.Show(SerialException.ToString());

Log_box.Text = Port_Name + "\r\n";

Log_box.Text += SerialException.ToString() + "\r\n";

Ready_icon.Visible = false;

COMport.Close();

}

catch (InvalidOperationException SerialException) // The specified port on the current instance of the SerialPort is already open

{

MessageBox.Show(SerialException.ToString());

Log_box.Text = Port_Name + "\r\n";

Log_box.Text += SerialException.ToString() + "\r\n";

Ready_icon.Visible = false;

COMport.Close();

}

catch //Any other ERROR

{

MessageBox.Show("Error in opening serial port - unknown error");

Ready_icon.Visible = false;

COMport.Close();

}

#endregion

}

if (COMport.IsOpen == true)

{

COMport.Write("acw:");

Log_box.Text += "Moving 1 degree anticlockwise" + "\r\n";

}

else

{

MessageBox.Show("Unable to communicate with serial port");

COMport.Close();

}

}

}

}

for (int x = 0; x < 420; x++ )//return to zero position

{//420 steps was found to be the correct value from when SR04 detects object

digitalWrite(pul, LOW);

delayMicroseconds(1000);

digitalWrite(pul, HIGH);

delayMicroseconds(100);

}

}

void takeReading()

{

if (senddata == true)

{

String tosend = String(pos) + "\n";

Serial.print(tosend);//sends motor position to serial port followed by line end

}

}

long SR04Distance()

{

digitalWrite(trig, LOW);//trigs a pulse to be transmitted from sensor

delayMicroseconds(2);

digitalWrite(trig, HIGH);

delayMicroseconds(10);

digitalWrite(trig, LOW);

long duration = pulseIn(echo, HIGH);//measures time before pulse returns

long d = duration / 58.2;//converts time into distance

delay(1);

return d;

}
